# Analyzing the performance of fluorescence parameters in the monitoring of leaf nitrogen content of paddy rice

**DOI:** 10.1038/srep28787

**Published:** 2016-06-28

**Authors:** Jian Yang, Wei Gong, Shuo Shi, Lin Du, Jia Sun, Shalei Song, Biwu Chen, Zhenbing Zhang

**Affiliations:** 1 State Key Laboratory of Information Engineering in Surveying, Mapping and Remote Sensing, Wuhan University, Wuhan, Hubei 430079, China; 2 Collaborative Innovation Center of Geospatial Technology, Wuhan, Hubei 430079, China; 3 School of Physics and Technology, Wuhan University, Wuhan, Hubei 430072, China; 4 Wuhan Institute of Physics and Mathematics, Chinese Academy of Sciences, Wuhan, Hubei 430071, China

## Abstract

Leaf nitrogen content (LNC) is a significant factor which can be utilized to monitor the status of paddy rice and it requires a reliable approach for fast and precise quantification. This investigation aims to quantitatively analyze the correlation between fluorescence parameters and LNC based on laser-induced fluorescence (LIF) technology. The fluorescence parameters exhibited a consistent positive linear correlation with LNC in different growing years (2014 and 2015) and different rice cultivars. The *R*^2^ of the models varied from 0.6978 to 0.9045. Support vector machine (SVM) was then utilized to verify the feasibility of the fluorescence parameters for monitoring LNC. Comparison of the fluorescence parameters indicated that F740 is the most sensitive (the *R*^2^ of linear regression analysis of the between predicted and measured values changed from 0.8475 to 0.9226, and REs ranged from 3.52% to 4.83%) to the changes in LNC among all fluorescence parameters. Experimental results demonstrated that fluorescence parameters based on LIF technology combined with SVM is a potential method for realizing real-time, non-destructive monitoring of paddy rice LNC, which can provide guidance for the decision-making of farmers in their N fertilization strategies.

Paddy rice plays an important role in the structure of the ecosystem. China grows paddy rice on approximately 30 million hectares per year and is the world’s leading producer of paddy rice^1^. Investigations have demonstrated that nitrogen (N) is an indispensable nutrient for the growth and development of cereal crops and is one of the most expensive, given that commercial N fertilizers represent the major cost in crop production. At present, China accounts for only 7% of the world’s cultivated land but accounts for 35% of the world’s N fertilizer consumption. Although the application of N fertilizers increases crop yields, increased use of N fertilizers affects ozone layer depletion and the global N cycle and also causes nitrate leaching problems in soil^2^,^3^. In addition, flooded paddy rice systems emit both N_2_O and CH_4_. The results of a recent meta-analysis showed that CH_4_ emissions from paddy rice systems account for almost 90% of the total global warming potential^1^,^4^,^5^. Therefore, precise leaf N content (LNC) detection is a promising strategy to obtain balance between N fertilization dose and cereal crop N needs in both time and space^6^,^7^.

In previous studies, many models have been proposed to analyze the LNC of plants based on passive remote sensing technology^7^,^8^,^9^. These methods are founded on the reflectance spectra of crops, which are related to leaf chlorophyll concentration (LCC) and the ability of photosynthesis. Numerous researchers analyzed and established the relationship between hyperspectral canopy reflectance and leaf nitrogen status (LNS) in crops to determine the characteristic spectral bands and key spectral parameters^8^,^10^. They reported that LNS can be estimated by using vegetation indexes through reliable regression equations established. However, passive remote sensing is restricted by many factors, such as weather situation, and measured time. To overcome these limitations, multi-wavelength canopy light detection and ranging (MWCL) was devised for remote sensing of vegetation reflection^11^. This technology has been widely utilized to monitor the nutrient stress of plants and classify different plant species^12^,^13^. It has also been utilized to estimate the biomass of vegetation^14^.

A few decades ago, the laser induced fluorescence (LIF) technique was proposed to study the growth status of plants^15^. When plants absorb the energy of a specific wavelength, a part of it vanishes by light emission at longer wavelengths within a few or dozens of nanoseconds; this process is called fluorescence emission^9^. With its advantages of rapidity, non-destructiveness, and no-preprocessing, LIF technology has been utilized to monitor N fertilization levels in crops^16^. Research has shown that a certain positive correlation exists between different N levels and fluorescence indicators and has demonstrated that LIF technology can be employed to detect the nutrient stress of crops. Thus, this technology has elicited an increasing amount of attention from researchers in the field of remote sensing^17^,^18^,^19^,^20^,^21^,^22^,^23^. Numerous investigations utilized fluorescence technology to analyze the N status of triticale, soybean, paddy rice, and cotton^24^,^25^,^26^. These investigations revealed that leaf fluorescence parameters can be implemented to estimate the LNC of crops.

These investigations mainly utilized fluorescence kinetics, minimum fluorescence intensity after short dark adaptation (F_o_), maximum fluorescence intensity after short dark adaptation (F_m_), and quantum efficiency of photosystem II^9^ as the indicators of the nutrient stress of crops. In addition, these studies discussed the effect of different nutrient stress values on fluorescence parameters in wheat, corn, and spring triticale^9^,^27^. In addition, our previous investigations demonstrated that LIF technology can be utilized to monitor the LNC of paddy rice. However, studies on fluorescence intensity and fluorescence ratios combined with algorithms to quantitatively evaluate the LNC of paddy rice based on LIF technology remain lacking. In addition, relevant studies that used LIF technology to analyze the LNC of different paddy rice cultivars in different growing years are also scarce. Thus, the main objectives of this investigation are as follows: (1) to discuss the influence of different paddy rice cultivars and growing years on the relationship between fluorescence parameters (fluorescence intensity and fluorescence ratio) and LNC and (2) to compare the performance of fluorescence parameters with the help of support vector machine (SVM) to quantitatively evaluate LNC in paddy rice.

## Results

### Fluorescence spectra

**Figure 1:**
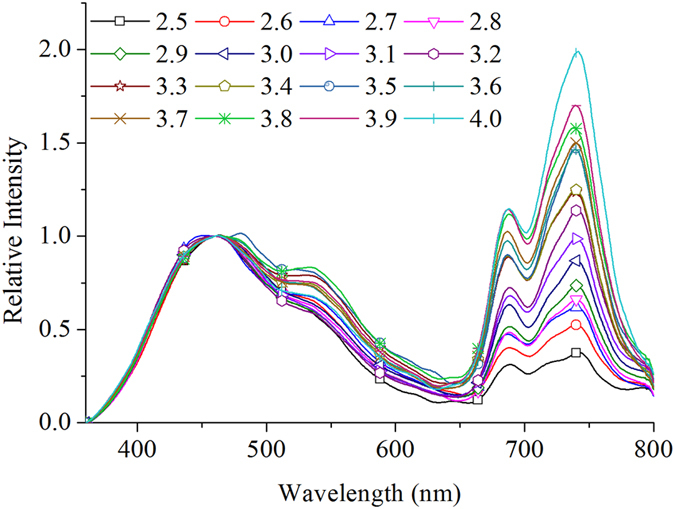
Normalized fluorescence spectra of paddy rice leaf (normalized to 1 at the 460 nm) with different LNCs (the LNCs range from 2.5 mg/g to 4.0 mg/g).

The fluorescence spectrum was measured with the LIF system. As shown in Fig. 1, all fluorescence spectra were normalized to 1 at 460 nm. These fluorescence spectra exhibited three main fluorescence peaks at 460, 685, and 740 nm, and a peak shoulder at 525 nm. According to Chappelle *et al*.^28^, the fluorescence peaks at 685 and 740 nm are attributed to chlorophyll a and b, respectively. Nicotinamide adenine dinucleotide (NADPH) and riboflavin are responsible for the fluorescence peak at 460 nm and the peak shoulder at 525 nm. According to previous investigations^16^,^29^,^30^,^31^, LNC is closely related to the fluorescence peaks (685 and 740 nm). Figure 1 shows that the intensity of the fluorescence peaks displayed a significant difference with the increase of LNC. with Compared with the studies of McMurtrey *et al*.^16^, fluorescence spectra exhibited a similar changing tendency in this study. Therefore, LIF technology can be employed to monitor the alterations of LNC.

### Analysis of the fluorescence parameters

**Figure 2:**
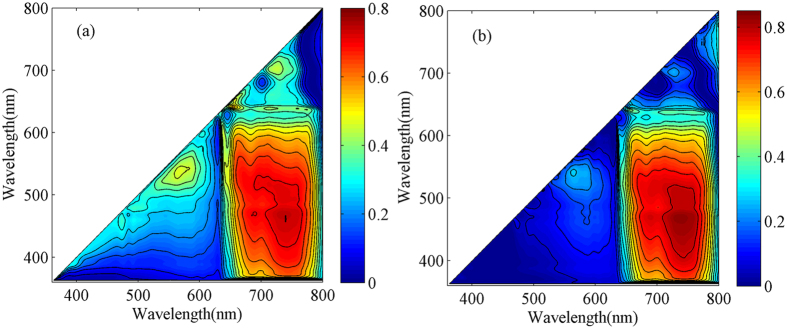
Contour maps for coefficients of determination (*R*^2^) between paddy rice leaf nitrogen content and fluorescence ratios in different growing years. 2014 (n = 324) (**a**); 2015 (n = 216) (**b**).

To analyze the optimal fluorescence emission wavelength for predicting paddy rice LNC, further discussion was conducted on the correlation between fluorescence ratios and LNC by using datasets from 2014 and 2015 (Fig. 2). Figure 2 was the contour maps of R^2^ between fluorescence ratios and LNC with the two wavelengths on the abscissa and the vertical axis. An overview of the statistical consequence for all fluorescence ratios also provided. As shown in Fig. 2, the correlation between fluorescence emission wavelengths at fluorescence peaks (center bands at around 685 and 740 nm) and LNC displayed a higher R^2^ than that of other wavelength bands. Thus, the main fluorescence peaks (F685: fluorescence intensity at 685 nm, and F740: fluorescence intensity at 740 nm) and fluorescence ratio (F740/F685: fluorescence intensity at 740 nm divided by that at 685 nm) were extracted to analyze the LNC of paddy rice.

### Relationship of fluorescence parameters to paddy rice LNC

**Figure 3:**
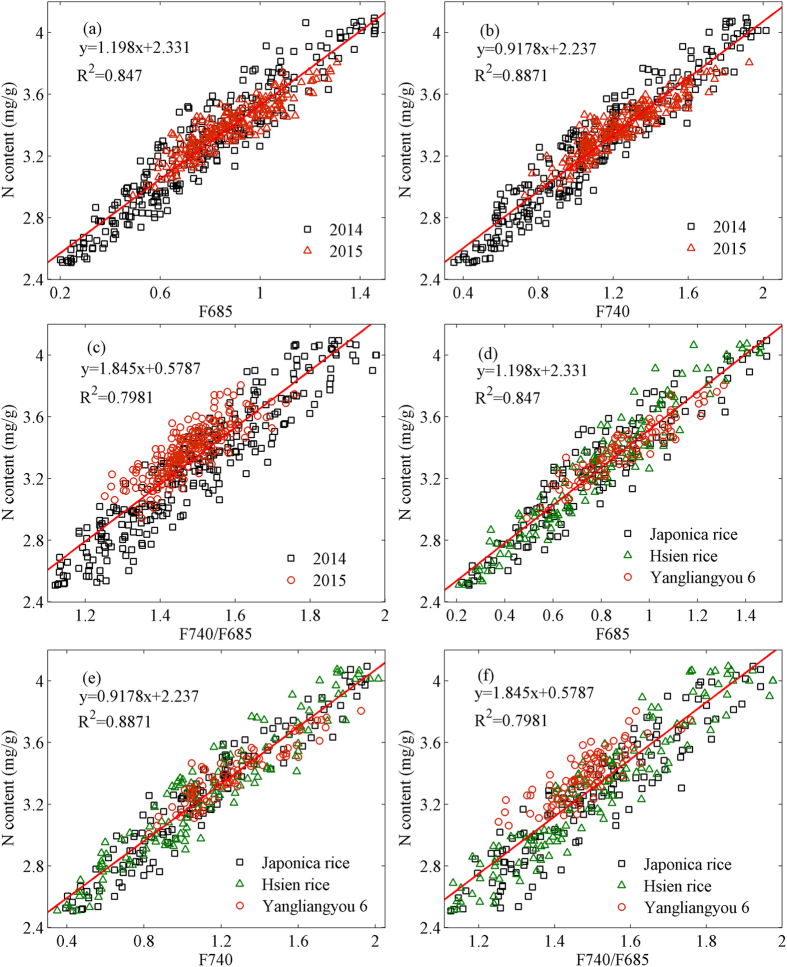
The relationships between fluorescence parameters (F685 (**a,d**); F740 (**b,e**); F740/F685 (**c,f**)) and paddy rice leaf nitrogen content (growing seasons (**a–c**); cultivars (**d–f**)). The red solid line denotes the regressive line for all experimental data.

As shown in Fig. 1, different LNCs result in different fluorescence characteristics at fluorescence peaks (685 and 740 nm). The intensity of fluorescence peaks (F685 and F740) increases with the creasing of LNC. Based on the analysis of fluorescence parameters and the previous investigations^16^,^26^,^29^,^30^, fluorescence parameters F740, F685, and F740/F685 were employed to analyze and inverse the LNC in this study. Thus, the linear correlation between these fluorescence parameters and LNC was established (Fig. 3).

**Figure 4:**
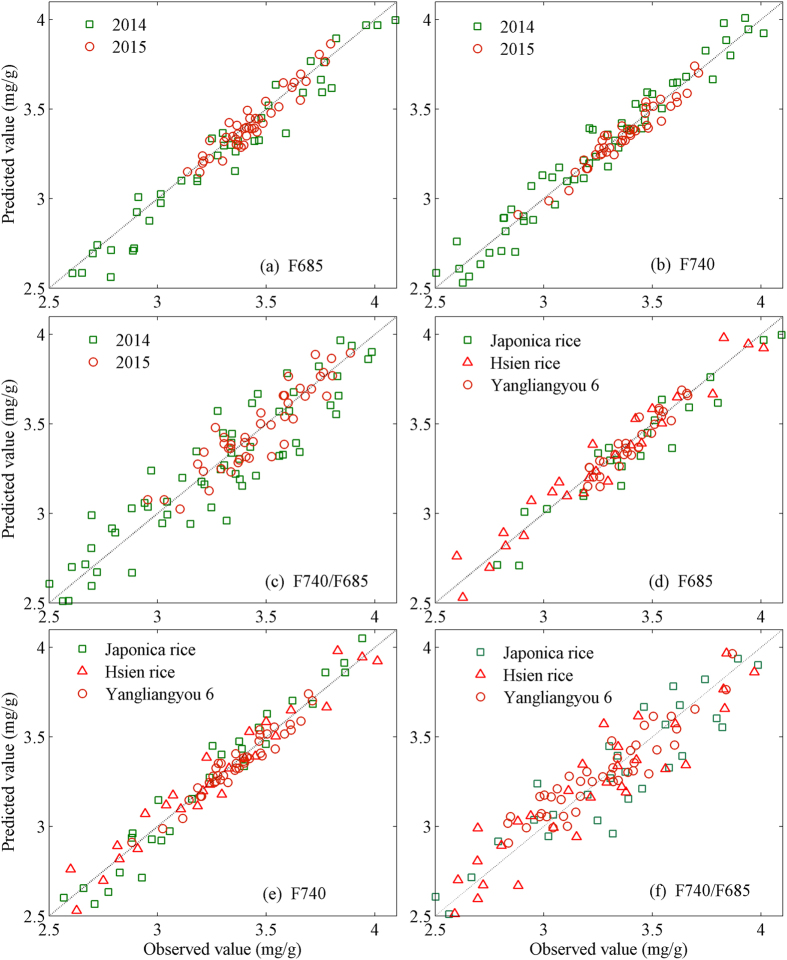
The relationship between the predicted LNC by using SVM based on different fluorescence parameters (F685 (**a,d**); F740 (**b,e**); F740/F685 (**c,f**)) and observed LNC for the three paddy rice cultivars and two growing seasons. The dotted line denotes the 1:1 line.

The fluorescence parameters (F685, F740, and F740/F685) displayed a closely positive linear correlation with LNC for different paddy rice cultivars and different growing seasons. For the two growing seasons (2014 and 2015), the *R*^2^ of the regression models of F685, F740, and F740/F685 areis 0.847, 0.8871, and 0.7981, respectively (Fig. 4(a–c)). They also display the similar results for the three paddy rice cultivars (Fig. 4(d–f)). To comprehensively analyze the relationship between the fluorescence parameters and LNC, a quantitative linear regression analysis was conducted on between fluorescence parameters and LNC for per growing season and per cultivar. The linear regression equations, *R*^2^ and RMSE of the fluorescence parameters and LNC are listed in Table 1.

The results demonstrated that the relationship between fluorescence parameters and LNC displayed high consistency in all the samples for different growing years and different paddy rice cultivars. For the three cultivars and the two growing years, the *R*^2^ of F685 ranges from 0.7811 to 0.8658, the *R*^2^ of F740 ranges from 0.8362 to 0.9045, and the *R*^2^ of F740/F685 ranges from 0.6978 to 0.842. Figure 3 and Table 1 show that the fluorescence indices (F685 and F740) have a higher correlation with LNC than that the fluorescence ratio (F740/F685), probably because F740/F685 is sensitive to the changes of leaf chlorophyll content and insensitive to the N content of plants^9^,^32^. However, the fluorescence parameters (F685, F740, and F740/F685) may be still useful and can be employed to accurately inverse the LNC of paddy rice.

### Inversion of LNC

To verify the possibilities that the fluorescence parameters (F685, F740 and F740/F685) can be used to precisely estimate LNC, SVM was applied in this study. The fluorescence spectra of each cultivar and each growing season were randomly divided into two parts: 70% was employed to train the SVM model on the basis of the different fluorescence parameters and 30% was utilized as a validation set to predict the LNC of paddy rice. For different cultivars and different growing years, the relationships between the predicted and observed paddy rice LNC are illustrated in Fig. 4.

As shown in Fig. 4, the LNC of paddy rice can be accurately predicted by utilizing these fluorescence parameters (F685, F740 and F740/F685) combined with SVM. The inversion results of all cultivars and growing years are consistent, and the predicted and measured LNCs are nearly in accordance with the line of 1:1 (the dotted line in Fig. 4). In addition, these results also demonstrate that the predicted LNC based on fluorescence intensity (F740 and F685) display a higher in accordance with the line of 1:1 (Fig. 4(a,b,d,e)) than that on the basis of the fluorescence ratio (F740/F685) (Fig. 4(c,f)). In order to analyze the accuracy and precision of the predicted LNC, the R2, RMSE and RE of linear regression analysis of the inversed LNC were listed in Table 2 in detail.

Table 2 shows that LNC can be accurately inversed by utilizing fluorescence parameters combined with SVM. The R^2^ (ranges from 0.8529 to 0.9226) of the relationship between the predicted LNC on the basis of the F740 and measured LNC is the highest among these fluorescence parameters. The R^2^ (ranges from 0.7842 to 0.8524) of F740/F685 is lower than that of the other fluorescence indices. The corresponding RMSEs are converse. These results demonstrate that fluorescence parameter F685 is more sensitive to the changes of LNC than F740/F685 and is lower than that F740. However, these fluorescence parameters are useful for inversing the LNC of paddy rice (all REs are below 10% in this research).

## Discussion

At present, LIF spectral data have already been employed to monitor the status of plants but only a few articles focused on paddy rice, despite its significance in environmental problems and its role as an aliment source^3^. A large number of investigations have also been conducted on the fluorescence of plants^17^,^33^ and summarized detailedly by Kalaji *et al*.^34^. This study aims to quantitatively estimate the LNC of paddy rice in different growing years and with different cultivars by using fluorescence parameters (F685, F740 and F740/F685) combined with SVM, which is a supervised learning algorithm. Thus, experiments on three cultivars of paddy rice were conducted in Central China Plain in 2014 and 2015.

Fig. 1 shows that the fluorescence spectra (ranging from 650 nm to 800 nm) increase with increased LNC. The reason is that the chlorophyll content in leaves would degrade and decrease rapidly and lutein then turns into a major pigment component when the LNC of the crop is reduced to threshold levels, which in turn affect the fluorescence characteristics of leaves^10^,^32^. Thus, Rademacher and Tartachnyk^35^ reported that LIF techniques that induce fluorescence intensity are more beneficial to remote and large-area field measurements than variable fluorescence measurement technologies^9^. The results of the current study demonstrate that F740 is more sensitive (*R*^2^ ranges from 0.8729 to 0.9426 for the three cultivars and two growing years) to the changes of LNC than the other two fluorescence parameters (F685 and F740/F685) in this study (Fig. 4 and Table 2). A probable interpretation is that the fluorescence in red-peak (around 685 nm) overlaps with the chlorophyll pigment absorption spectrum, and re-absorption depends on the chlorophyll content of thylakoid membranes in a leaf^36^,^37^. In addition, Brestic *et al*.^38^ analyzed the correlation between F740/F685 and basal fluorescence (F_o_), non-photochemical quenching (NPQ) and demonstrated that F740/F685 is sensitive also to changes in photosystem I to photosystem II ratio and the difference of LNC will result in the change of F740/F685 ratio.

Most fluorescence kinetics and variable fluorescence parameters were proposed to monitor pigment content and were conductive to the monitoring of the photosynthetic efficiency of plants^19^,^39^,^40^,^41^. However, perhaps they are not sensitive enough to monitor the N content of crops^29^,^42^. Meanwhile, fluorescence kinetics indices for estimating N status are mostly based on a specific fluorescence emission wavelength band, and their inherent disadvantages limit their application in evaluating LNC^32^. Compared with fluorescence kinetics indices, the fluorescence parameters in this study (F740, F685, and F740/F685), which were utilized to inverse LNC in paddy rice, exhibited higher accuracy and precision (REs < 10%). They also displayed a certain reliability in inversing LNC, given that the experimental data included three paddy rice cultivars (Japonica rice, Hsien rice and Yangliangyou 6) and two growing years (2014 and 2015).

Although this investigation preformed with two growing years and three cultivars of paddy rice, the monitoring models of LNC based on LIF technology were established and verified by using SVM in an ecological region with a typical subtropical monsoon climate. However, other significant fluorescence parameters and the regression analysis models still need to be analyzed comprehensively in other ecological locations, including different growing years and production systems. To obtain the optimal algorithm and fluorescence parameters, LIF technology combined with other multivariate analyses also needs to be tested and used to estimate the LNC of other crops in the future research.

## Conclusions

In this research, linear regression models between different fluorescence parameters (F685, F740 and F740/F685) and LNC were established. A close linear positive correlation was observed between fluorescence parameters and LNC for different paddy rice cultivars (Japonica rice, Hsien rice, and Yangliangyou 6) and different growing years (2014 and 2015). The *R*^2^ of linear regression analysis obtained by using the fluorescence parameters ranged from 0.6378 to 0.9145. SVM was utilized to verify if LNC can be evaluated on the basis of the fluorescence parameters (F685, F740, and F740/F685). The inversion results revealed that the N content of paddy rice can be accurately evaluated by utilizing the fluorescence parameters combined with SVM. In addition, F740 is the most sensitive to the changes of the LNC of paddy rice among these fluorescence parameters. Therefore, these fluorescence parameters combined with SVM will be very helpful in realizing real-time, non-destructive monitoring of paddy rice leaf N status based on LIF technology. They can also be employed to guide farmers in the application of reasonable doses of N fertilizers, which may result in remarkable environmental and economic interests because of reduced environmental pollution and increased N utilization efficiency.

## Materials and Methods

### Study areas and site description

All experiments were conducted at Junchuan County, Suizhou City, in the province of Hubei, and in the experimental station of Huazhong Agricultural University (HAU) in Wuhan City in the Jianghan China Plain during the paddy rice cultivating seasons of 2014 and 2015. The experimental area is characterized by a typical subtropical monsoon climate with abundant rainfall; it is sunny and hot in summer and cold in winter. The longitude of this area ranges from 113°41′ E to 115°05′ E and the latitude varies from 29°58′ N to 31°22′ N. The sunshine duration and rainfall are above 1800 hours and over 1200 mm per year, respectively. Thus, the area is suitable for growing paddy rice and is also known as one of the largest agricultural production installations for providing food security in China^43^.

The paddy rice varieties cultivated were Japonica rice and Hsien rice. They were cultivated in Junchuan County, Suizhou City, in the province of Hubei, China. There cultivars were seeded on April 27 and then transplanted on June 1, 2014. During the entire growth period, six N fertilization levels of urea were used (0, 189, 229.5, 270, 310.5, and 351 kg/ha) in the experimental fields. N fertilization was divided into four splits: 30% at seeding, 20% at tillering, 25% at shooting, and 25% at booting. The experimental field had an absolute block design with three replications for each treatment under the same cultivation conditions. Other managements were advised by the local farm extension service in rice production. Paddy rice samples were collected on July 15 and August 1, 2014.

Yangliangyou 6 was cultivated in the experimental station of HAU in Wuhan City in 2015. It was seeded on April 30 and transplanted on May 27. During the entire growth period, four N fertilization levels of urea were used (0, 120, 180, and 240 kg/ha) in the experimental fields. N fertilization was divided into three splits: 60% at seeding, 20% at tillering, and 20% at shooting. The experimental field had an absolute block design with three replications for each treatment. Paddy rice samples were gathered on four dates (July 20, 22, 24, and 26, 2015).

### Measurement of Fluorescence spectrum

The system of LIF consisted with three parts: the excitation assembly, optical receiver system, and the data collection and treatment part. The laser source was an Nd: YAG that emitted pulses with the output energy and the pulse duration time being 1.5* *mJ and 5 ns respectively, and the omitted wavelength was 355 nm with a pulse repetition frequency of 20* *Hz. A single-mode optical fiber with a diameter of 200 μm and 25^o^ angular field of view was utilized to transmit the fluorescence signal which was placed at the position of the focus of the Maksutov-Cassegrain telescope. In addition, a long-pass filter of 355 nm (the edge of 360 nm) which was used to eliminate the reflected light from the laser entering the optical fiber was positioned behind the telescope. The fluorescence induced by the ultraviolet laser then entered the spectrometer. The fluorescence signal was measured by utilizing the ICCD, and the data was stored in a personal computer. In this study, the fluorescence spectra ranged from 360 to 800 nm with sampling interval of 0.5 nm. Normalized fluorescence intensity (normalized to 1 at the 460 nm) varying with the wavelength was showed in Fig. 1.

### Measurement of leaf Nitrogen content

Paddy rice leaves were destructively sampled by stochastically cutting six leaves with three replicates for each experimental field. All samples were fully expanded the second leaves from the top. These samples were sealed in plastic bags, kept in an ice chest, and then immediately transported to the laboratory for fluorescence spectra measurements^43^. All samples were immediately sent to Wuhan Academy of Agricultural Science and Technology for measurement of LNC after fluorescence measurements. The Keldjahl method was utilized to determine the paddy rice LNC in this study^44^,^45^.

### Analytical method

SVM displays excellent generalization performance in practical application with a solid theoretical foundation in statistical learning theory^46^. SVM has the ability to construct both linear and nonlinear inversion and can be used in heterogeneous classes with small samples. Therefore, SVM has been successfully implemented in a wide range of pattern recognition issues^46^. The detailed description of SVM can be found in references^46^,^47^. As one of the most accurate and robust machine learning methods, SVM was then utilized in this investigation to verify the possibility of LIF parameters quantitatively inversing the LNC. The correlation between fluorescence parameters and LNC was analyzed with MATLAB 2009a. Before analysis, wavelet transform was applied to eliminate the spectral noises. Moving window polynomial fitting was then applied to smooth the fluorescence spectra. All fluorescence spectra were then randomly divided into two sections: 70% as the training set for SVM model and 30% as the validation set to predict LNC. The performance of the model was analyzed by comparing the differences in the coefficient of determination (R^2^), root mean square error (RMSE), and relative error (RE) in prediction. RMSE and RE can be presented as follows:


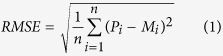



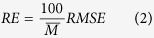


where *M*_i_ denotes measured values, *P*_i_ represents predicted values, and *n* corresponds to the number of samples. 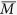 denotes the mean measured values. RE displays the relative differences between the predicted and observed values. A high *R*^2^ and low RMSE and RE denote the high precision and accuracy of the model in predicting LNC. The prediction is recognized as excellent if RE is less than 10%, good if RE ranges from 10% to 20%, fair if RE ranges from 20% to 30%, and poor if RE >30%^10^,^48^,^49^.

## Additional Information

**How to cite this article**: Yang, J. *et al*. Analyzing the performance of fluorescence parameters in the monitoring of leaf nitrogen content of paddy rice. *Sci. Rep.* **6**, 28787; doi: 10.1038/srep28787 (2016).

## Figures and Tables

**Table 1:** Quantitative relationship of paddy rice leaf nitrogen content (*y*) to individual fluorescence parameter (*x*) for the three paddy rice cultivars and two growing years.

		F685 vs. LNC	F740 vs. LNC	F740/F685 vs. LNC
Years	2014			
	Eq.	y = 1.271x + 2.256	y = 0.953x + 2.189	y = 1.93x + 0.362
	RMSE (mg/g)	0.1244	0.1186	0.1427
	* R*^2^	0.8658	0.9045	0.842
	2015			
	Eq.	y = 0.908x + 2.586	y = 0.711x + 2.497	y = 1.53x + 1.126
	RMSE (mg/g)	0.07206	0.0671	0.09697
	* R*^2^	0.7811	0.8362	0.6978
Cultivars	Japonica rice			
	Eq.	y = 1.241x + 2.277	y = 0.923x + 2.18	y = 1.916x + 0.365
	RMSE (mg/g)	0.1336	0.1122	0.1475
	* R*^2^	0.8301	0.8925	0.806
	Hsien rice			
	Eq.	y = 1.295x + 2.241	y = 0.94x + 2.207	y = 1.93x + 0.378
	RMSE (mg/g)	0.1132	0.1277	0.1389
	* R*^2^	0.8421	0.8635	0.825
	Yangliangyou 6			
	Eq.	y = 0.94x + 2.56	y = 0.707x + 2.488	y = 1.474x + 1.216
	RMSE (mg/g)	0.07462	0.06211	0.09027
	* R*^2^	0.8084	0.8415	0.7239

**Table 2:** Linear regression analysis between the predicted using SVM and observed LNC for the three cultivars and two growing years based on the different fluorescence parameters (F685, F740, and F740/F685).

		F685 vs. LNC	F740 vs. LNC	F740/F685 vs. LNC
Years	2014			
	* R*^2^	0.8852	0.9226	0.8524
	RMSE (mg/g)	0.1088	0.085	0.1538
	RE (%)	4.29	3.63	5.74
	2015			
	* R*^2^	0.8482	0.8629	0.7842
	RMSE (mg/g)	0.1353	0.0647	0.0943
	RE (%)	5.95	4.53	7.72
Cultivars	Japonica rice			
	* R*^2^	0.8607	0.9094	0.8232
	RMSE (mg/g)	0.1034	0.091	0.1617
	RE (%)	4.13	3.81	6.94
	Hsien rice			
	* R*^2^	0.8788	0.8941	0.8412
	RMSE (mg/g)	0.0913	0.1081	0.1508
	RE (%)	3.91	3.52	5.69
	Yangliangyou 6			
	* R*^2^	0.8249	0.8529	0.8064
	RMSE (mg/g)	0.1297	0.1049	0.1876
	RE (%)	5.46	4.83	8.08
